# Cross-Platform Analysis of Endothelial Transcriptomic Changes Associated with Diabetes

**DOI:** 10.21203/rs.3.rs-10781628/v1

**Published:** 2026-09-08

**Authors:** Yingjun Luo, Kailing Huang, Jiawei Sun, Meirigeng Qi, Xiaofang Tang, Zhen Bouman Chen

**Affiliations:** City of Hope; City of Hope; City of Hope; City of Hope; City of Hope; City of Hope

**Keywords:** Endothelial dysfunction, RNA-seq, Type 2 Diabetes Mellitus, Mesenteric artery, Endothelial heterogeneity, Angiogenesis

## Abstract

Endothelial dysfunction is an early hallmark of diabetes and a key driver of diabetic vascular complications. However, the transcriptional responses of endothelial cells (ECs) to diabetic stress across different vascular beds and the extent to which cultured ECs recapitulate these changes remain incompletely understood. Here, we profiled transcriptomes of mesenteric arterial intima from non-diabetic, pre-diabetic, and type 2 diabetic donors and compared diabetes-associated differentially expressed genes with those identified in five cultured human EC subtypes, including umbilical vein, aortic, dermal microvascular, lymphatic microvascular, and liver sinusoidal ECs, exposed to high glucose and TNF-a. Human arterial intima and cultured ECs shared multiple diabetes-associated stress-response and vascular remodeling programs, with metabolic alterations more prominent in human donor ECs and inflammatory and immune pathways more pronounced in cultured ECs. Despite these context-dependent differences, we identified a subset of genes, including *GGT5*, *HTR1B*, *SLC25A34-AS1*, and *LINC01235*, consistently downregulated in both human donor samples and multiple cultured EC subtypes under diabetic conditions. Functional analyses demonstrated that suppression of these genes impaired angiogenesis while promoting endothelial inflammation and senescence, recapitulating key features of endothelial dysfunction. Together, our findings reveal both shared and context-dependent endothelial responses to diabetes and provide a transcriptomic resource for studying disease-associated EC changes and identifying candidate regulators of diabetes-associated endothelial dysfunction.

## Introduction

Diabetes mellitus (DM) is a global epidemic affecting over 580 million adults worldwide, with Type 2 Diabetes Mellitus (T2D) accounting for over 90% of all cases^1^. Individuals with T2D are at a substantially increased risk of cardiovascular disease, including coronary artery disease, heart failure, and peripheral artery disease^2–5^. These complications arise from combined effects of chronic hyperglycemia, hypertension, dyslipidemia, and systemic inflammation. Central to these pathological processes is the vascular endothelium, a dynamic and metabolically active monolayer lining the luminal surface of blood vessels. Positioned at the interface between circulating blood and the vessel wall, endothelial cells (ECs) regulate vascular tone, thrombosis, leukocyte trafficking, barrier integrity, and metabolic homeostasis^6,7^. EC dysfunction is an early feature of diabetes that often precedes overt hyperglycemia and plays a critical role in the development of both metabolic and cardiovascular complications.

Accumulating evidence demonstrates that ECs exhibit remarkable heterogeneity across vascular beds. ECs from different tissues/organs possess distinct structural, functional, and transcriptomic characteristics shaped by local microenvironment, resulting in highly context-dependent responses to physiological and pathophysiological stimuli^8–10^. These transcriptional programs include both protein-coding genes (PCGs) and non-coding RNAs, including long non-coding RNAs (lncRNAs) that have emerged as important regulators of endothelial function and diabetic vascular complications^11–13^. Despite this heterogeneity, most studies of endothelial response to diabetic stress have relied on cultured primary ECs^14,15^. Human umbilical vein ECs (HUVECs), in particular, have been widely used and have been instrumental in advancing vascular biology, but they do not fully recapitulate the heterogeneity of ECs across vascular beds^16,17^. Culture-based systems nevertheless provide an accessible and controllable platform for dissecting endothelial mechanisms. However, ECs maintained in the static culture can progressively lose aspects of their native identity and properties, due to the absence of physiological flow, cell interactions, and tissue-specific microenvironments, and thus may not retain faithfully their original gene expression and natural response to disease conditions^18,19^.

These limitations underscore the need to characterize endothelial transcriptional responses directly in relevant human vascular tissues, particularly in the context of T2D^20,21^. Here, we profiled endothelial transcriptomes from freshly isolated human mesenteric arteries obtained from non-diabetic (ND), pre-diabetic (PD), and T2D donors. Using a lumen-flushing technique^22,23^, we isolated intimal RNA from intact arteries, enabling transcriptomic profiling of ECs in their native, minimally perturbed state using RNA-sequencing (RNA-seq). We identified differentially expressed genes (DEGs) and pathways associated with diabetes progression in human arterial endothelium and compared these signatures with transcriptomic responses of five cultured EC subtypes exposed to high glucose and TNF-a. We defined shared and context-dependent endothelial responses to diabetic stress, providing a transcriptomic resource for evaluating experimental EC models, and identified novel candidate genes and pathways underlying diabetic endothelial dysfunction and cardiometabolic diseases.

## Results

### Transcriptomic profiling of human mesenteric arterial intima from donors of various diabetic states

We profiled the transcriptome of human mesenteric arterial intima from a total of 22 donors, including 7 non-diabetic [ND; average glycated hemoglobin (HbA1c) = 5.3%; all men; 1 Caucasian, 4 Hispanic, 2 Asian], 6 pre-diabetic (PD; average HbA1c = 5.8%; 4 men and 2 women; 1 Caucasian, 4 Hispanic, 1 Black) and 9 diabetic (T2D; average HbA1c = 8.0%; 6 men and 3 women; 4 Caucasian, 4 Hispanic, 1 Black) using RNA-seq. These donors were selected based on their demographic and biometric information and medical history, with comparable biological ages among three groups (**Supplementary Fig. S1A** and **Supplementary Table S1**).

Using the GRCh38 human reference genome with Ensembl release 104 annotation, we identified 52,279 genes across the 22 samples. To determine whether global gene expression profiles distinguished the predefined donor groups, we performed partial least squares discriminant analysis (PLS-DA), a supervised dimensionality reduction and classification method^24,25^. Using either all detected genes or the DEGs identified through one-way Analysis of Variance (ANOVA, *P* value < 0.05), PLS-DA clearly separated 3 groups of donors (Fig. 1A, B). Among the total of 762 DEGs, 321 were PCGs (42.1%), 274 were lncRNAs (36%), and 150 were pseudogenes (19.7%) (Fig. 1C). These genes exhibited distinct expression patterns across three groups of donors (Fig. 1D).

The 762 DEGs were enriched in metabolic and endothelial pathways associated with diabetic pathology, including glucose metabolism, endoplasmic reticulum stress, insulin signaling, extracellular matrix (ECM) organization, and hemostasis (Fig. 1E). We further identified 215 DEGs whose expression correlated with HbA1c levels (**Supplementary Fig. S1B**). These genes also clearly separated ND, PD, and T2D donors, exhibited a similar gene composition as all detected genes and showed stronger enrichment for pathways related to glucose metabolism, insulin function, and vascular signaling (**Supplementary Fig. S1, C-E**).

To identify early transcriptional changes associated with diabetes, we identified genes that were significantly altered in PD and remained similarly dysregulated in T2D (Fig. 2A, B). While DEGs downregulated at the PD stage were enriched in pathways regulating cell cycle, cell-cell adhesion, DNA repair, and leukocyte migration and trafficking (Fig. 2A), those upregulated were enriched in pathways related to diabetic cardiomyopathy, molecular transport and secretion, and immune responses (Fig. 2B). These data suggest that at the prediabetic stage, the arterial endothelium is already shifting toward stress-, inflammatory-, and metabolic disease-associated programs.

By comparison, genes exhibiting significant changes only in T2D but not in PD, likely represent transcriptional alterations associated with later-stage disease progression. These DEGs showed strong enrichment in metabolic processes, including lipid, branched-chain amino acid, and protein metabolism, as well as immune pathways involving leukocyte differentiation, lymphocyte-mediated immunity, and chemotaxis. Pathways related to wound responses also emerged at this stage (Fig. 2C, D). These data suggest that as diabetes progresses, endothelial transcriptional dysregulation expands to encompass broader metabolic remodeling, immune activation, and impaired vascular repair.

### Transcriptomic profiling of cultured human ECs subjected to HT

We exploited the in vitro model of hyperglycemia and inflammation associated with T2D by treating 5 EC subtypes with high glucose (25 mM) and TNF-a (5 ng/mL) (HT) for 24 hours. These ECs include human umbilical vein endothelial cells (HUVECs), human aortic endothelial cells (HAECs), human dermal microvascular endothelial cells (HMVECs), human dermal lymphatic microvascular endothelial cells (LMVECs), and human liver sinusoidal endothelial cells (LSECs). Using the same genome annotation, we identified 37,000–40,000 genes in each of these EC subtypes.

Principal component analysis (PCA) based on all detected genes revealed clear separation among five EC subtypes. While HUVECs, HAECs, and LMVECs clustered closely together, HMVECs and LSECs displayed more distinct transcriptomic profiles (Fig. 3A). The HT treatment induced clear transcriptional shift across all EC subtypes, evidenced by PCA based on DEGs (|log_2_ fold change [FC]|) > 1, p_adj_<0.05) (Fig. 3B). The separation between control (NM, 25 mM mannitol) and HT-treated cells was more pronounced in HUVECs, HAECs, and LMVECs than in HMVECs and LSECs (Fig. 3B), consistent with the greater numbers of HT-induced DEGs in HUVECs (1,131), HAECs (1,956), and LMVECs (1,304) than in HMVECs (409) and LSECs (467) (Fig. 3C), suggesting HUVECs, HAECs, and LMVECs may be more responsive to HT treatment.

Pathway enrichment analysis of DEGs in various EC subtypes showed that HT altered similar pathways in HUVECs, HAECs and LMVECs, including immune and cytokine signaling, inflammatory responses, stimulus/stress responses as top pathways, followed by cell migration, adhesion and proliferation. DEGs in HMVECs were also enriched in these pathways but to a lesser extent. In contrast, LSECs displayed a more restricted transcriptional response, with enrichment largely confined to metabolic reprogramming, stress and differentiation pathways (Fig. 3D). Notably, cellular response to lipids was exclusively enriched in HMVECs and LSECs. Across all five EC subtypes, we identified 6 common DEGs, i.e., *BCL2A1*, *CCL5*, *CD74*, *CSF2*, *STARD10* and *TNIP3*, all of which were upregulated by HT (**Supplementary Table S2**).

Among the HT-responsive genes, 132 DEGs were shared across four subtypes (Fig. 3C, **Supplementary Table S2**), including key regulators of EC function *NOS3* and *VCAM1*, which were downregulated and upregulated by HT, respectively. Similarly, established markers of EC dysfunction and inflammatory activation *ICAM1*, *FN1* and *CCL2*, were increased by HT in at least four EC subtypes (**Supplementary Fig. S2**). We previously identified four HT-responsive lncRNAs, *LINC01235*, *LINC01013, LINC00607* and *LINC02154* using single-cell RNA-seq (scRNA-seq) of HUVECs^26^. These lncRNAs exhibited greater EC-subtype-specific regulation: *LINC01235* was downregulated by HT in all EC subtypes except LSECs, while *LINC01013*, *LINC00607* and *LINC02154* showed variable responses across EC subtypes (**Supplementary Fig. S2**). These findings are consistent with the greater cell- and tissue-specificity of lncRNA expression compared with PCGs^27–29^.

### Candidate genes shared between diabetic human arterial intima and HT-treated cultured ECs

To identify DM-associated genes in human mesenteric arteries that are also responsive to HT in cultured ECs, we first intersected 762 DEGs identified in the intima by ANOVA across three donor groups with 1,272 HT-responsive DEGs shared by at least 2 EC subtypes (Fig. 4A, left arm). This analysis identified 16 genes, comprising 12 PCGs and 4 lncRNAs (**Supplementary Table S3**). Among these, 4 PCGs (*TCF7*, *PSAT1*, *RAB42*, *HTR1B*) and two partially annotated lncRNAs (*ENSG00000289029* and *ENSG00000289161*) showed concordant expression changes between human mesenteric intima and at least two subtypes of cultured ECs.

Given the limited number of candidates identified, we next employed an expanded screening strategy to capture additional potentially relevant genes (Fig. 4A, right arm). For the human mesenteric intima, we combined DEGs from ANOVA across the three donor groups, ANOVA comparing ND vs. (PD+T2D), and DESeq2 analysis comparing ND vs. T2D, yielding a total of 2,731 genes. For cultured EC datasets, we applied different criteria for PCGs and lncRNAs given that lncRNAs generally exhibit greater tissue and cell-type specificity and often more modest differential expression than PCGs^27–30^. For PCGs, we selected HT-responsive genes with |log_2_ FC| > 1 in at least four EC subtypes, whereas for lncRNAs, we selected genes with |log_2_ FC| > 0.5 in at least two EC subtypes. This approach identified 54 genes shared between the human and cultured EC datasets, comprising 13 PCGs and 41 lncRNAs. Of these, 3 PCGs (*AK5*, *HTR1B*, *GGT5*) and 20 lncRNAs exhibited concordant expression changes between human mesenteric intima and cultured ECs, with HUVEC or HAEC expressions prioritized when responses differed across EC subtypes (Fig. 4A, **Supplementary Table S4**). Six of the concordant lncRNAs were annotated: *LINC01089*, *FRMD6-AS2*, *BCL10-AS1*, *PM20D1-AS1*, *SLC25A34-AS1*, and *LINC01235*.

Combining the candidates identified by the two approaches yielded 26 DEGs showing concordant changes, of which 12 clearly annotated genes (6 PCG and 6 lncRNAs) were selected for further evaluation, whose expression patterns across cultured EC subtypes and human mesenteric intima are shown in Fig. 4B and C. To further prioritize candidates with consistent responses to vascular stress, we examined two independent RNA-seq datasets of HUVECs exposed to IL-1β + TGFβ2^31^ or hypoxia^32^, stimuli relevant to EC dysfunction and vascular disease (**Supplementary Fig. S3A, B**). *AK5*, *HTR1B*, *GGT5*, *SLC25A34-AS1* and *LINC01235* were consistently downregulated across all pathological conditions. We next examined their tissue distribution using Genotype Tissue Expression (GTEx) data^33^. Among these five genes, *HTR1B* and *GGT5*, *SLC25A34-AS1* and *LINC01235*, showed preferential expression in human arteries (Fig. 4A, **Supplementary Fig. S3C**). Thus, we prioritized these four genes as candidate regulators of diabetic endothelial dysfunction. Using HAECs, which share an arterial origin as the mesenteric arterial intima, we further validated the HT suppression of these four genes (Fig. 4D).

#### Functional validation of GGT5, HTR1B, SLC25A34-AS1, and LINC01235 in HAECs

The four prioritized candidates represent distinct pathways potentially relevant to endothelial homeostasis but remain incompletely characterized in diabetic vascular dysfunction. *HTR1B* encodes the Gi/o-coupled serotonin receptor 5-HT1B. Although serotonin signaling has been implicated in endothelial angiogenesis^34,35^, whether *HTR1B* contributes to endothelial homeostasis in diabetes remains unclear. *GGT5* encodes a γ-glutamyl transferase involved in extracellular glutathione metabolism and redox homeostasis^36–38^. *GGT5* is downregulated in cerebrovascular ECs in patients with Alzheimer’s disease (AD) whereas endothelial overexpression of *GGT5* ameliorated AD-related pathologies in mouse models, suggesting a protective role for endothelial *GGT5*^39^. Whether *GGT5* regulates endothelial homeostasis in diabetes remains unknown. The two lncRNAs are even less characterized: *SLC25A34-AS1* is an antisense lncRNA with largely unknown biological functions, although its protein-coding counterpart, *SLC25A34*, encodes a mitochondrial carrier implicated in metabolic homeostasis^40^. *LINC01235* was among the most strongly dysregulated lncRNAs in HT-treated HUVECs in our previous study^26^ and has otherwise been studied primarily in cancer^41–43^, with recent evidence implicating its potential role in atherosclerosis^44^.

The limited characterization and consistent suppression of these 4 candidate genes under pathological conditions prompted functional investigation in ECs. To model their loss in diabetes, we used small interfering RNAs (siRNA) to knock down (KD) the PCGs *HTR1B* and *GGT5* and locked nucleic acid (LNA) GapmeRs to suppress the lncRNAs *SCL25A34-AS1* and *LINC01235* in HAECs (Fig. 5G). Specifically, for *SLC25A34-AS1*, we designed LNA that does not target the overlapping region with the host gene *SLC25A34*. Thus, the observed effects are unlikely to be attributed to a concurrent KD of *SLC25A34*. Although depletion of these genes did not induce overt cell death or morphological change, KD of each gene consistently impaired endothelial angiogenic function, as demonstrated by reduced 3D spheroid sprouting, 2D tube formation, and wound closure (Fig. 5A-F). Suppression of all 4 genes also increased monocyte adhesion (Fig. 5H, I) and senescence-associated (SA) b-gal staining (Fig. 5J, K). Thus, despite their distinct molecular identities, loss of each candidate converged on a shared dysfunctional endothelial phenotype characterized by impaired angiogenesis, enhanced inflammatory activation, and accelerated senescence.

## Discussion

Vascular endothelium plays a central role in cardiovascular homeostasis and disease, whereas studies performed with ECs directly isolated from human vessels are few. Beckman et al. profiled freshly isolated ECs from the cubital veins of patients with T2D^21^, providing important insights into diabetes-associated alterations in human venous ECs. More recently, we used scRNA-seq and spatial transcriptomics to characterize EC gene expression and EC-macrophages interactions in human mesenteric arteries^22,45^. Complementing these studies, the present work applies bulk RNA-seq, providing greater sequencing depth and sensitivity for detecting low-abundance and incompletely annotated transcripts. Importantly, we included donors with PD, enabling us to distinguish transcriptional alterations that emerge early during disease progression from those associated with established T2D. For example, *ANGPTL4*, *LRG1* and *SLC5A2*, genes linked to vascular and metabolic homeostasis, exhibited changes only in PD but not in T2D donors^46–48^, suggesting that PD may involve transient adaptive or compensatory endothelial responses that are lost or attenuated with disease progression. Alternatively, these differences may partly reflect medication or diet management in the T2D group. Such early-stage transcriptional changes may provide candidate biomarkers or therapeutic targets for intervening before overt DM-associated vascular dysfunction.

ECs derived from distinct vascular beds exhibit substantial differences in morphology, gene expression profiles, functional properties, and responses to physiological or pathological stimuli^18,20,49^. Although HUVECs have been widely used because of their accessibility and ease of culture, they originate from fetal tissue and may not accurately recapitulate the characteristics of adult or tissue-specific endothelium^50^. Accordingly, the importance of tissue-appropriate EC models is increasingly recognized, such as LSECs for liver disease and microvascular ECs for diseases affecting microcirculation. Previous studies have begun to define how EC origins influence responses to environmental stimuli. For example, Maurya et al. found that HAECs and HUVECs shared canonical shear stress-responsive programs but differed in the magnitude and temporal dynamics of their responses, with HAECs exhibiting an earlier transcriptional response to flow^51^. Jamil et al. demonstrated distinct transcriptomic and epigenetic signatures of LSECs compared with other EC^52^. More recently, single-cell transcriptomic studies revealed^20^.. By exposing five EC subtypes to identical HT conditions, our study provides a direct comparison of their transcriptional responses and reveals both common and subtype-specific changes associated with diabetic stress.

The 26 DEGs showing concordant changes between human mesenteric intima and cultured ECs represent a relatively small subset of the transcriptional responses detected in either system. A similarly limited overlap between the in vivo and in vitro endothelial response was reported previously when venous ECs from patients with T2D were compared with HUVECs exposed to high glucose^21^. Consistent with this context dependence, several canonical markers of endothelial function including *NOS3*, *VCAM1*, *ICAM1*, were not significantly altered in our human donor intima samples (**Supplementary Fig. 1F**). These differences likely reflect the distinct biological contexts captured by two platforms with human donor ECs integrating the complex effects of diabetes with comorbidities, risk factors, medications, and lifestyle interventions. Cultured ECs provide a controlled platform to investigate endothelial responses to defined diabetic stimuli, whereas native ECs from human vessels integrate the cumulative effects of chronic metabolic disturbances, cell interactions, ECM remodeling and the tissue microenvironment. In addition, although luminal flushing enriches the intimal fraction for ECs, minor contributions by smooth muscle cells and/or other adherent cell types cannot be completely excluded^23^. Thus, the two approaches should be viewed as complementary, with in vitro models facilitating mechanistic studies and human tissues providing physiological relevance. The concordant DEGs identified across both systems may therefore represent particularly robust endothelial responses that are conserved across experimental contexts.

We identified 4 artery-enriched genes that were consistently dysregulated in diabetic conditions. Despite their distinct molecular identities and putative functions, suppression of each gene in HAECs converged on a common dysfunctional endothelial phenotype, characterized by impaired angiogenic capacity, increased monocyte adhesion, and accelerated senescence. These changes recapitulate key features induced by HT and observed in diabetic vascular complications, supporting the possibility that these genes normally contribute to endothelial homeostasis and that their coordinated suppression may promote vascular dysfunction, inflammation, and impaired repair in diabetes. Of note, further KD of these genes under HT did not significantly increase monocyte adhesion (**Supplementary Fig. S4**). One possible explanation is that HT had already sufficiently suppressed their expression or downstream protective pathways, such that additional KD produced little further effect. Future studies should define how these and other candidate genes regulate endothelial function, determine whether their effects are additive or interconnected, and establish their functional and therapeutic relevance in diabetic vasculopathies.

## Methods

### Human tissues

Human tissue studies were conducted on deidentified specimens obtained from the Southern California Islet Cell Resource Center at City of Hope. Informed consent for the use of postmortem human tissues was obtained from the donors’ next of kin. Ethical approval for this study was granted by the Institutional Review Board of City of Hope (IRB #23648). All methods involving human tissues were performed in accordance with the relevant institutional guidelines and regulations and in accordance with the Declaration of Helsinki. T2D was identified based on diagnosis in the donors’ medical records as well as the percentage of glycated hemoglobin A1c (HbA1c) of 6.4% or higher. HbA1c of 5.7% to 6.4% was classified as prediabetes, and HbA1c below 5.7% is considered normal. Donor information is summarized in **Supplementary Table S1**.

### EC culture and treatment

For cultured ECs used for RNA-seq, HUVECs (Cell Applications Inc., Catalog # 200p-05n and Lot # 3363 and 2914), HMVECs (Cell Applications Inc., Catalog # 100-05n and Lot # 2747 and 2709), HAECs (Cell Applications Inc., Catalog # 304-05a, and Lot # 4493 and 7930), and LMVECs (Cell Applications Inc., Catalog # 100L-05a) between passages 3–4 were used; LSECs (ScienCell Research Laboratories, Catalog # 5000) between 2–3 passages were used. HMVECs, LMVECs, and LSECs were cultured in the corresponding medium recommended by the manufacturers. HUVECs and HAECs were cultured in M199 (Sigma M2520) supplemented with 10% and 20% FBS (Hyclone, SH30910.02) respectively, β-endothelial cell growth factor (Sigma E1388), and 100 units/mL penicillin and streptomycin (Thermo Fisher Scientific 15140-122). HMVECs and LMVECs were cultured in the corresponding medium recommended by the vendor. All the cells were maintained at 37°C with 5% CO_2_ in a hydrated incubator. The cells were treated with D-glucose (25 mM) + TNF-a (5 ng/mL; Thermo Fisher Scientific PHC3011) at around 60–70% confluency for 24 hours. The control cells were maintained in normal medium supplemented with 25 mM mannitol (NM) as an osmotic control. The siRNA (Sigma) or LNA (QIAGEN) was transfected into HAECs with Lipofectamine RNAiMAX (Thermo Fisher Scientific 13778150) following the protocol provided by the manufacturer, in the final concentration of 20 nM. The cells were cultured in M199 complete medium 4–6 hours after transfection. The functional assays were performed 48 hours after transfection. The sequences of siRNA and LNAs are listed in **Supplementary Table S5**.

### RNA isolation, qPCR and sequencing

For human mesenteric arteries, a piece of the second major branch of the abdominal aorta (2–8 cm in length, 0.3-1.0 cm in diameter) was obtained from the islet transplantation donors. The intimal RNA was isolated by quickly flushing the inner lumen once with TRIzol (Thermo Fisher Scientific 15596018) following an established method^22^. 100 ng total RNA per sample was subjected to library construction using QIAseq Stranded mRNA Select Kit (QIAGEN 180773) following the user manual. For in vitro EC RNA-seq libraries, RNA was extracted from cells using TRIzol following the manufacturer’s instructions. 1000 ng total RNA per sample was subjected to library construction using KAPA mRNA HyperPrep Kit (Roche Diagnostics KK8580) following the manufacturer’s manual. The libraries were sequenced in Illumina NovaSeq 6000 using the PE150 mode.

For qPCR, total RNA was extracted using TRIzol reagent and cDNAs were synthesized using the PrimeScript^™^ RT Master Mix (TaKaRa RR036A) containing both Oligo-dT and random hexamer primers. qPCR was performed with Taq Pro Universal SYBR qPCR Master Mix (Vazyme Q712-02) following the manufacturer’s protocol on the Bio-Rad CFX Connect Real Time system with β-actin as internal control. All primer sequences used for qPCR amplification are listed in **Supplementary Table S5**.

### RNA-seq data analysis

RNA-seq data were analyzed as previously described^26^. The FASTQ files were aligned to human reference genome (GRCh38. Ensembl 104) using HISAT2 (version 2.2.1)^53^. FeatureCounts from Subread package (version 2.12.0)^54^ were used to generate counts of the uniquely mapped reads. The raw count matrices were prefiltered to remove genes with very low read counts and used as input for differential gene expression analysis using the R package DESeq2 (version 1.36.0)^55^. DEGs of the in vitro ECs dataset by DESeq2 analysis were defined by |Log_2_ FC| > 1, adjusted *P* value < 0.05. To expand the list of DE lncRNAs, the cutoff was set to |Log_2_ FC| > 0.5, *P* value < 0.05. DEGs of the human donor samples were identified by either one-way ANOVA across the three groups or two groups (combining PD and T2D) using *P* value < 0.05 as cutoff; or by DESeq2 analysis using a cutoff |Log_2_ FC| > 1, *P* value < 0.05. Gene Ontology (GO) enrichment analysis was performed using the Gene Ontology Consortium platform^56^ and Benjamini-Hochberg corrected *P* values < 0.05 were considered significantly enriched pathways. To assess tissue distribution, expression levels of the candidate genes were retrieved from the GTEx database. Genes were defined as artery-enriched if expression in at least one arterial tissue ranked among the top ten tissues with the highest TPM values. To minimize the inclusion of genes with limited detectability in human samples for candidate screening, genes were required to have a median TPM > 0 in at least two of the three donor groups.

### Spheroid sprouting, tube formation, wound healing assays

The 3D spheroid sprouting assay, tube formation assay, and wound healing assay were performed as previously described^57^. Briefly, HAECs were plated on a Matrigel (Corning 356234) coated 24-well plate in M199 complete medium, incubated for up to 8 hours in 5% CO_2_ at 37°C, and examined for capillary tube formation under an inverted microscope and imaged. For the scratch assay, HAECs were seeded onto 24-well plates and grown to confluence. Cell monolayers were wounded with a 200 μL pipette tip to generate a cut of ~ 1 mm in width. After two washing steps, cells were incubated for 9 hours and imaged. For 3D spheroid sprouting assay, HAECs were trypsinized, counted and resuspended at 10^6^ cells/mL in M199 complete medium. 125 μL of the resuspended cells, 3.875 mL of M199 complete medium and 1 mL of 0.3% (W/V) Methylcellulose (MC) (Sigma M0512-100G) in M199 medium were mixed to bring the total volume to 5 mL and then 20 μL mixture was used to form one aggregate and incubated overnight. On the next day, cell aggregates were collected, washed, centrifuged, and resuspended in pre-chilled Matrigel (Corning 356234) to allow for two aggregates per 20 μL. After 30 minutes of incubation, the gelled aggregates were then overlaid with M199 complete medium containing 100 ng/mL of VEGF (Sigma V7259-10UG) and incubated for 3 days, with monitoring for sprouting.

### Monocyte adhesion and SA-β-galactosidase (SA-b-gal) assays

The monocyte adhesion assay and SA-b-gal staining were performed as previously described^26,58^. THP1 cells were labeled with CellTracker^™^ Green CMFDA Dye (Thermo Fisher Scientific C2925) and incubated with HAECs for 15–30 minutes. The nonattached cells were washed off with the M199 complete medium. The attached monocyte numbers were evaluated on BioTek Cytation^™^ 1 Cell Imaging Multi-Mode Reader (Agilent) using the green-fluorescent channel. Total numbers per sample were calculated from 3–5 randomly selected fields. HAECs (passage 8) were used for assay. Cytochemical staining for SA-b-gal was performed using the SA-b-gal Staining Kit (Cell Signaling Technology 9860S) following the manufacturer’s manual.

### Statistical analysis

For the in vitro RNA-seq experiments, 2 biological replicates were used. For other experiments, at least three independent experiments were performed. For these experiments, statistical analysis was performed using *t*-test (unpaired, two-sided) between two groups or one-way ANOVA followed by Dunnett’s test relative to the corresponding controls. *P* < 0.05 was considered statistically significant unless otherwise specified. The quantification and statistical analysis have also been specified and detailed in the results and figure legends.

## Supplementary Material

This is a list of supplementary files associated with this preprint. Click to download.

- Supplementarymaterialsfinal0821.pdf

## Figures and Tables

**Figure 1:**
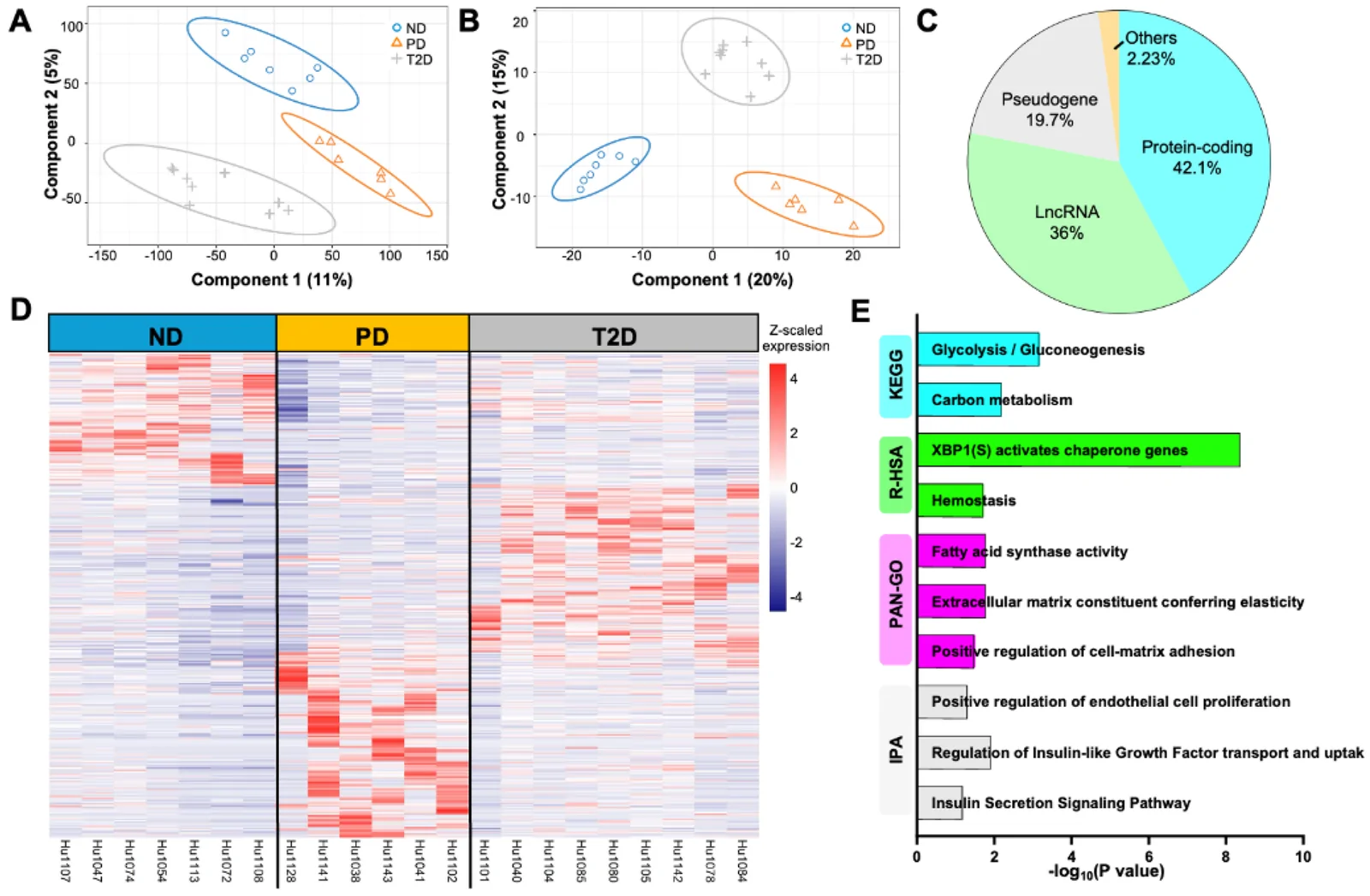
Global transcriptomic differences distinguish human arterial intima from donors of different diabetic states. **(A)** Partial least squares discriminant analysis (PLS-DA) based on all detectable genes in non-diabetic (ND), pre-diabetic (PD) and type 2 diabetic (T2D) donors. **(B)**PLS-DA based on the 762 differentially expressed genes (DEGs) identified by ANOVA analysis, demonstrating clear separation among the three donor groups. **(C)** Pie chart for categories of 762 DEGs. **(D)**Heatmap showing z-scaled expression of the 762 DEGs across the 22 donor samples. **(E)** Pathway enrichment analysis of 762 DEGs using KEGG (Kyoto Encyclopedia of Genes and Genomes), R-HSA (Human Reactome), PAN-GO (PAN-GO Functionome), IPA (Ingenuity Pathway Analysis), ranked by the *P* values.

**Figure 2:**
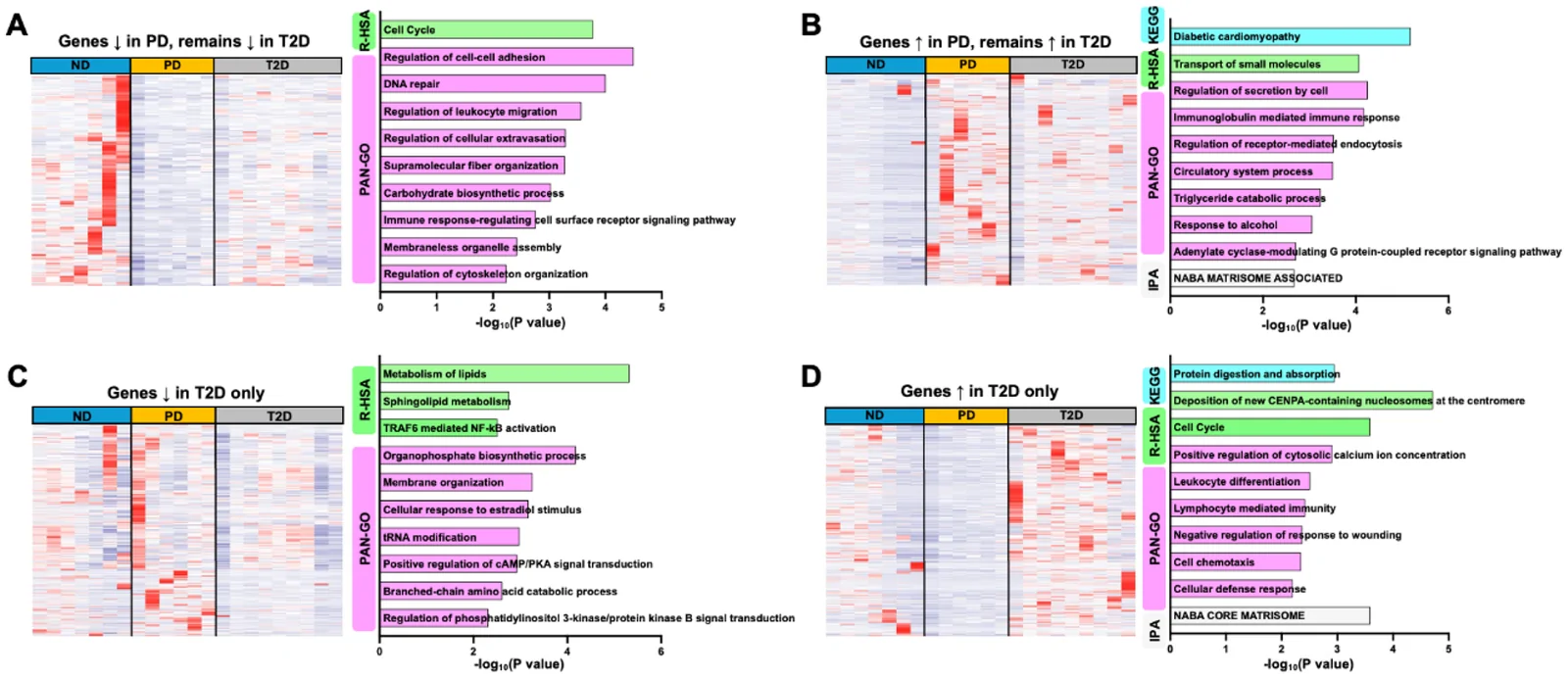
Stage-specific DEG signatures associated with pre-diabetes and diabetic stages in the human mesenteric arterial intima. **(A)** Expression patterns and enriched pathways of downregulated genes only at PD stage. **(B)** Expression patterns and enriched pathways of upregulated genes only at PD stage. **(C)** Expression patterns and enriched pathways of downregulated genes only at diabetic stage. **(D)** Expression patterns and enriched pathways of upregulated genes only at diabetic stage. Pathway annotations from different resources were ranked by *P* values.

**Figure 3:**
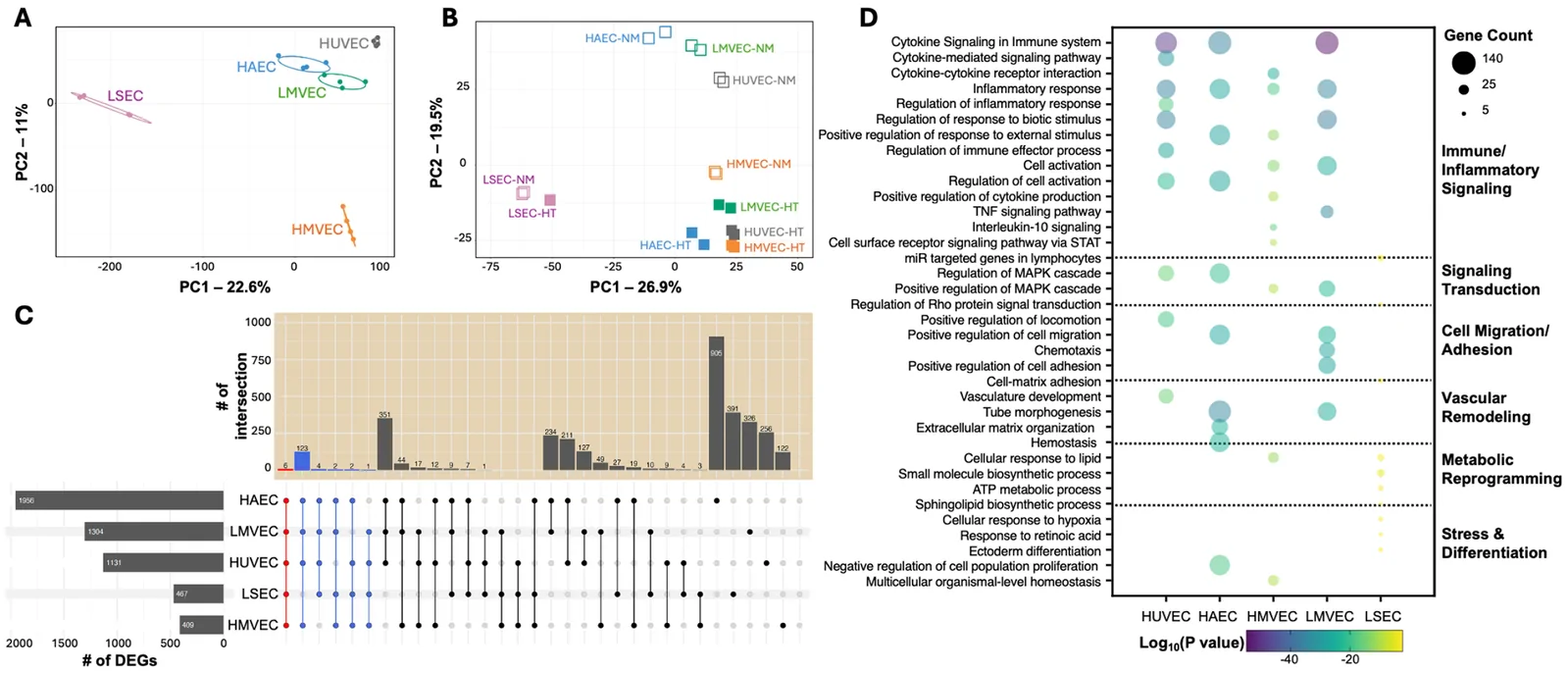
Transcriptomic responses to a combined treatment of high glucose and TNF-a (HT) in EC subtypes. (**A)** Principal component analysis (PCA) based on all detected genes showing transcriptomic variation among the 5 EC subtypes. **(B)** PCA based on DEGs (HT vs. NM) in each subtype showing separation between NM- and HT-treated samples. **(C)** Numbers of DEGs identified in each EC subtype, including common and subtype-specific DEGs. **(D)** Pathway enrichment analysis of DEGs in each EC subtype. Dot color indicates *P* value, and dot size represents the number of genes.

**Figure 4:**
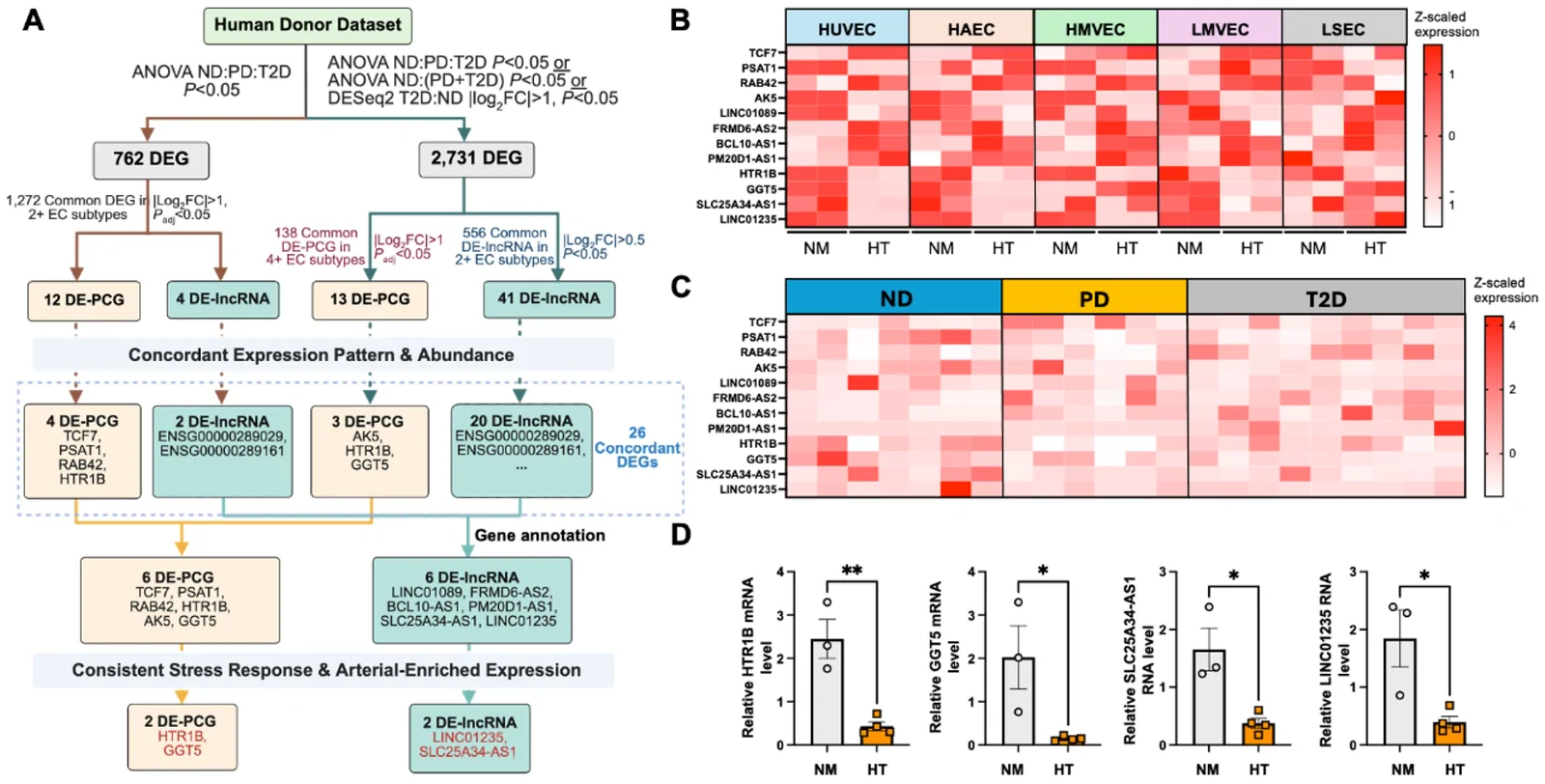
Identification of candidate genes shared between diabetic human arterial intima and HT-treated cultured ECs. **(A)** Workflow illustrating the identification of DEGs shared between human mesenteric arterial intima and cultured ECs exposed to HT and selection of candidate genes for functional validation (created using BioRender.com). **(B)** Heatmap showing representative common DEGs across five EC subtypes, plotted with z-scaled expression. **(C)** Heatmap showing the expression of the corresponding DEGs in human donor samples, plotted with z-scaled expression. **(D)** Validation of 4 selected DEGs in HT-treated HAECs by quantitative PCR. Data are represented as mean ± SEM. *P < 0.05 based on unpaired t-tests between NM (n=3) and HT (n=4).

**Figure 5:**
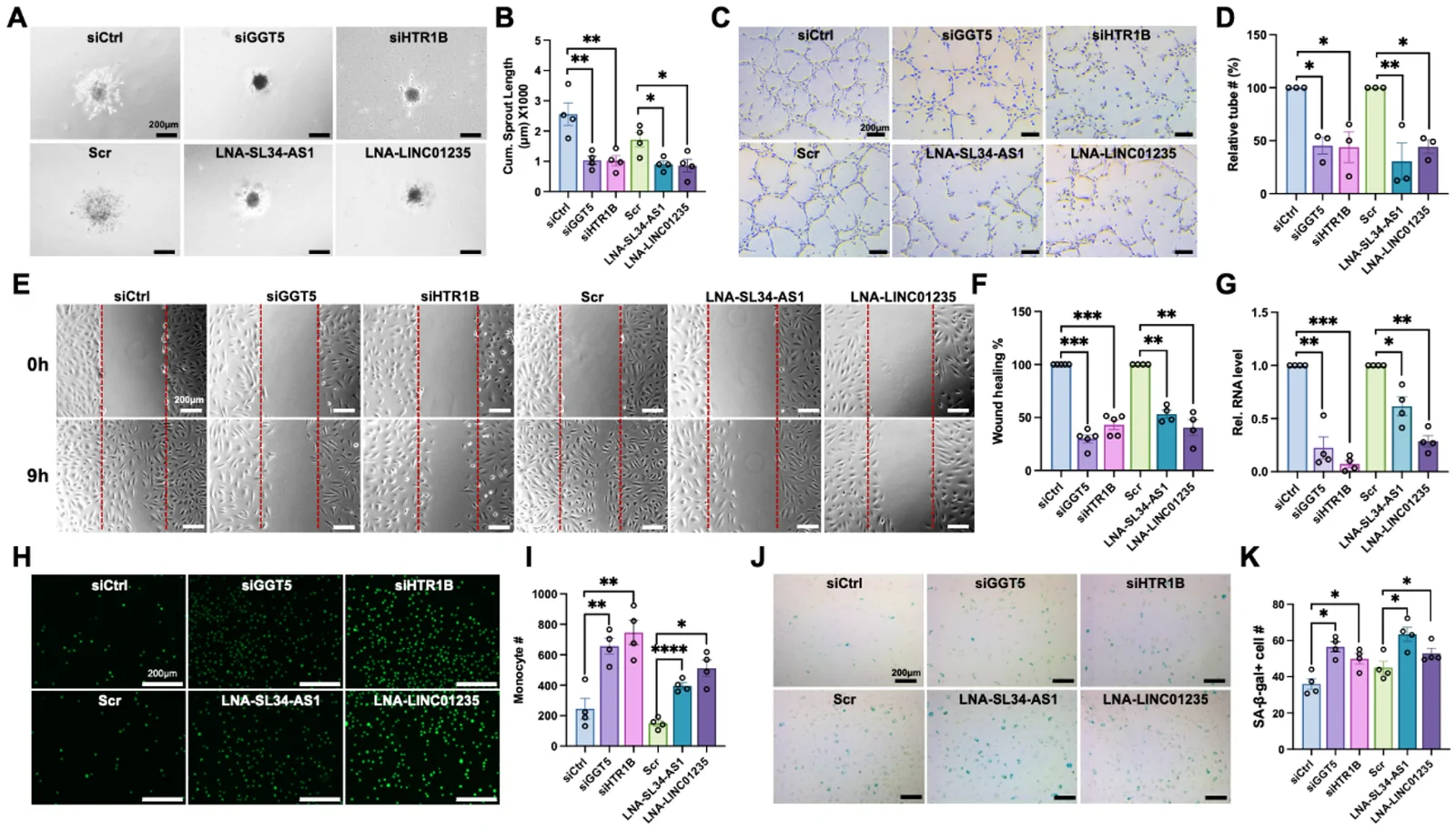
Functional characterization of GGT5, HTR1B, SLC25A34-AS1, and LINC01235 in HAEC. ECs transfected with indicated small interfering RNAs (siRNA) or locked nucleic acid (LNA) GapmeRs were used in spheroid sprouting **(A and B)**, tube formation **(C and D)**, scratch **(E and F)**, and monocyte adhesion assays **(H and I)** and β-gal staining **(J and K)**. Representative images of sprouting spheroids **(A)**, the tube formation **(C)**, the wound closure **(E)**, the attachment of fluorescently labeled THP-1 cells (**H)** and the positively β-gal-stained cells **(J).** The sprout length **(B)**, total tube numbers **(D)**, wound closure % **(F)**, the number of monocytes attached to ECs **(I)**, and positively β-gal-stained cell numbers **(K)** were quantified. The tube number or wound closure area in control siRNA (siCtrl) or scramble LNA GapmeR (Scr) were set to 100%. **(G)** DEG KD efficiency in HAECs. The relative RNA level of each gene targeted was normalized to the corresponding control (set as 1). siGGT5 and siHTR1B, siRNAs targeting GGT5 and HTR1B, respectively. LNA-SL34-AS1 and LNA-LINC01235, LNA GapmeRs targeting SLC25A34-AS1 and LINC01235, respectively. Data are represented as mean ± SEM from at least three independent experiments. *P < 0.05, ** *P* < 0.01, **P < 0.001, ****P < 0.0001, based on one-way ANOVA followed by Dunnett’s test relative to the corresponding controls in B, D, F, G, I and K. Scale bar = 200 μm**.**

## Data Availability

All high-throughput sequencing data generated by this study have been deposited on GEO (accession No. GSE341403). RNA-seq datasets from HUVECs treated with IL-1β and TGFβ2, and HUVECs treated with normoxia or hypoxia originated from NCBI GEO, accession No. GSE118446 and GSE70330, respectively. The GEO dataset (GSE341403) can be accessed for review using the following direct link: https://www.ncbi.nlm.nih.gov/geo/query/acc.cgi?acc=GSE341403 with this reviewer token: mdqzoaegdbmbfyh.
